# History, Structure and Agency in Global Health Governance: Comment on "Global Health Governance Challenges 2016 – Are We Ready?"

**DOI:** 10.15171/ijhpm.2016.119

**Published:** 2016-08-29

**Authors:** Stephen Gill, Solomon R. Benatar

**Affiliations:** ^1^Department of Political Science, York University, Toronto, ON, Canada.; ^2^University of Cape Town, Cape Town, South Africa.; ^3^Dalla Lana School of Public Health, University of Toronto, Toronto, ON, Canada.

**Keywords:** Political Power, Structure, Agency, Global Health Governance

## Abstract

Ilona Kickbusch’s thought provoking editorial is criticized in this commentary, partly because she fails to refer to previous critical work on the global conditions and policies that sustain inequality, poverty, poor health and damage to the biosphere and, as a result, she misreads global power and elides consideration of the fundamental historical structures of political and material power that shape agency in global health governance. We also doubt that global health can be improved through structures and processes of multilateralism that are premised on the continued reproduction of the ecologically myopic and socially unsustainable market civilization model of capitalist development that currently prevails in the world economy. This model drives net financial flows from poor to rich countries and from the poor to the affluent and super wealthy individuals. By contrast, we suggest that significant progress in global health requires a profound and socially just restructuring of global power, greater global solidarity and the "development of sustainability."

Ilona Kickbusch’s important editorial suggests that a global power shift is occurring, involving “the rise of the rest” relative to the United States, and with it, changes in the nature of multilateralism. This may mean that 2016 could be a turning point towards improved global health and better global health governance.^1^ Her points of departure are: (1) The failures of our development models to improve health, to narrow disparities and to attend to the most vulnerable populations in fragile states; (2) Inadequacy of current global health governing mechanisms, and (3) Some optimism arising from new resolve for improving global health associated with recent economic advances in low- and middle-income countries and from resolutions emanating from international conferences. We are in agreement with her first two points but we are critical of her article on several grounds.

## Failure to Acknowledge Previous Analyses

First, she fails to acknowledge a wide range of work that has analysed and critiqued global structures and the specific failings to which she refers – her most distant reference is to 2004. Consideration of previous work might help to better reveal some of the fundamental realities of power that have shaped and continue to shape global health governance. At least since the 1970s the adverse impacts of human activities on the environment,^2-4^ global health,^5-7^ the instability of populations who are becoming refugees,^8^ the (im)morality of the global economy with its adverse effects on democracy^9^ and the multiple crises that threaten life in general, have all been critically addressed.^10,11^

For example, in 1980 Johan Galtung emphasised the significance of the underlying structures of power that configure the possibilities for action or agency in global affairs*:*

*“There is a crisis in the world today, now felt even by those of us who enjoy the power and privilege at the top of the world. There is a crisis of violence …. There is a crisis of misery, and threat of poverty. There is a crisis of repression, and threat of repression of all human rights. There is a crisis in the environment …. At the root of the crises is not resource scarcity or price increases or population pressure, but the world structure.”* ^12^

Much has changed since Galtung wrote. The world has become much more capitalist and unequal following the collapse of the Soviet Bloc, and threats to the biosphere have multiplied and accelerated, particularly since China has increasingly become integrated into the now dominant market-based, but increasingly crisis-prone, capitalist accumulation structures. The contemporary magnitude of global crises has been reflected in some recent books.^13,14^

In this context, we would argue that current frameworks of multilateralism are ultimately constituted and shaped by the fundamental economic and political structures, forces and knowledge frameworks that configure the global political economy, namely those of actually existing neoliberal capitalism. Prevailing frameworks of global governance are premised upon a dominant energy-intensive neoliberal *market civilization model of development*, in a global system ultimately protected by the geopolitical power of the United States and its principal allies.^15^ The market civilization model is, however, contradictory: whilst it is premised upon individualism and the realisation of self through consumerism, it rests upon a socially unsustainable and ecologically myopic idea of “consume today and forget about the future.”^16^ This model depends upon acceleration in energy-intensive production and distribution patterns that increase the depletion of non-renewable resources and generate vast quantities of pollution and waste. By definition the market civilization model is socially exclusive and tends to predominantly benefit an affluent minority of the world’s population that consumes a growing proportion of global resources and services, whereas billions of people are deemed to be disposable or are expelled from the world economy as largely irrelevant.^17^ At issue, therefore, in this contradictory world order is the struggle for reasonable conditions of existence amid mechanisms and developments that are endangering the health of us all and of the planet.

Whilst there is no single cause for the developments just outlined, we have argued recently that it is undeniable that some of the principal historical forces involved are connected to the structures and processes associated with the specific model of development that prevails in capitalism today.^16^ This neoliberal market civilization model involves structural acceleration in processes within the world economy that together produce contradictions inimical to the general health of populations and the sustainability of the biosphere. Whilst we cannot go into detail on any of these points, it is worth listing some of them here. They include (1) Intensification of the exploitation of human beings, social processes, and nature for purposes of profit; (2) Incremental dispossession of communities of their basic and local means of subsistence and livelihood; (3) Acceleration in the turnover time of the production and sale of commodities to generate quicker accumulation of profits for firms and investors; and (4) Restructuring or privatization of previously public institutions and public goods, including provisions for healthcare and education. As the latter become increasingly subject to market forces and values, they are treated like commodities that can be simply bought and sold for reasons of profit.^18^ All the above go with greater inequality and social dislocation as well as pollution, toxicity, planned obsolescence, waste and relatively uncontrolled despoliation of the environment – at the expense of conditions for healthy living, and for delivery of healthcare.

In the case of health, the forms of power/knowledge that define agendas for policy and research are shaped by the power of large corporations and governments premised upon governance through mechanisms associated with market forces. In global health, this specifically means large pharmaceutical corporations, well-capitalized privately oriented philanthropies such as the Wellcome Trust or the Gates Foundation, private medical insurance firms and a related academic/health science complex. A market-driven system structures the nature of the research and training of medical and health personnel. The system tends to focus principally on therapies for afflictions that yield profit – the diseases of the affluent, or those who are able to pay for the treatment. It is increasingly associated with private ownership/control over medical education and research, healthcare provision, insurance and the provision of drugs and therapies. This pattern is reflected in the work of the Consortium of Universities on Global Health, configured by a biomedical model of disease that pays little or no attention to powerful causal structures that generate poor health, a model oriented towards the health of individuals as opposed to that of whole populations.

We might add here that capitalist enterprises are not focused on the promotion of the global health of the world’s population for its own sake, but rather on the accumulation of capital via the profit system. Thus, capital can profit from obesity as well as from hunger, and will only invest its resources in markets that have the capacity to buy the goods and services that it is able to supply. In a capitalist market system, prices and incomes determine if one eats or starves: the poor go hungry. This contradiction partly helps to explain why we are now living in a world where 25% of the world’s population is obese and 25% of the world’s population is starving or virtually starving,^19^ and wide disparities in health and longevity persist.^20^

So what do these developments reflect? Simply put (neo-liberal) capitalism is not just a set of economic or market-based processes but also a (contradictory and highly unequal) *system of power.* This should not be confused with a power shift from the United States towards “the rest.” Rather the shift or more accurately, the redistribution of social and economic power, is from the poor- and middle-classes to the wealthy, both in the United States, the BRICS (Brazil, Russia, India, China, and South Africa) and much of the rest of the world.

This is why in today’s global political economy recent patterns of accumulation generate a situation in 2016 where a mere 62 super-rich people hold more wealth than half of the world’s people (3.6 billion people); the top 1% now own more than the other 99% of the world’s people combined. Since 2010 the wealth of the poorest half of the world’s people has dropped by 38% (by one trillion dollars). “Meanwhile the wealth of the richest 62 (9 were women) has increased by more than half a trillion dollars to $1.76 trillion.”^21^ Indeed, the plutocracy’s share of global wealth has been massively amplified since the 2008 global financial crash, partly because any losses they sustained were socialised by governments bailing them out, and subsequently they could take advantage of very cheap loans in the loose monetary conditions provided by central banks in response to the crisis, enabling them to make much more money.^22^ One indicator of the priorities of this system of global governance since 2008, therefore, is the gigantic sums made available to bailout large capitalist enterprises (estimates for the United States, United Kingdom, and European Union (EU) between 2008-2013 vary between $17 trillion and $40 trillion) in response to the possibility of a meltdown of global capitalism. These sums massively outweighed the relatively very small and now diminishing amounts dedicated to the transfer of aid to poor countries and for the Millennium Development Goals (MDGs) (MDGs were replaced in 2016 by the Sustainable Development Goals).

Nevertheless, the policy choices just noted need to be assessed in terms of what economists call their “opportunity costs,” namely the alternatives that are foregone in favouring some policies over others. As noted above, much of the enormous transfer of material resources since the crash of 2008 has been carried out through monetary policies – which have only indirect effects on general economic conditions. By contrast fiscal policy, which has much more direct, focused and immediate effects, might have been targeted to build new hospitals, schools, infrastructure, daycare facilities, renewable energy resources, and other socially and ecologically beneficial investments as well as to fund-related activities and jobs. This would have had the effect of not only improving health and social conditions but also creating jobs, and injecting liquidity into the economy in ways that would help to regenerate cleaner and more socially responsive patterns of economic growth. It would have addressed some of the problems associated with the global financial and economic crisis and deteriorating provisions for healthcare. However, fiscal policy (specifically expenditures) has been relatively under-utilized in response to the crisis, reflected in policies of fiscal austerity that have been used in much of the world.

Moreover, the opportunity costs of opting to favour monetary policy are rarely publicly debated. This is despite the fact that monetary policy tends to transfer resources principally to financial institutions and to those firms and individuals who are able to access large amounts of credit (loans), which by definition largely excludes the majority of society. Lest this point be misunderstood as simply the view of critics of neoliberal capitalism, we can cite evidence provided by one of the principal beneficiaries of the recent monetary expansion, wealthy financial capitalist George Soros. He demystified some of this policy choice and its effects in a speech at the 2015 World Economic Forum in Davos, Switzerland. Soros pointed out that the continued use of monetary policies would “increase inequality between rich and poor both in regards of the countries and people.” He further argued that, “excessive reliance on monetary policy tends to enrich the owners of property and at the same time will not relieve the downward pressure on wages.”^23^ On a global basis, this policy choice, widely adopted across the G-20, reflects what Stanley Druckenmiller, the billionaire hedge fund manager (and former associate of Soros) pronounced was “The biggest redistribution of wealth from the middle-class and the poor to the rich ever.”^24^

The priorities of global governance are, therefore, very clear and it would be surprising if those priorities did not influence the agenda for the various conferences and initiatives as well as the material provisions allocated to and informing the global governance of health.

In a nutshell, global governance frameworks are still dominated by the wealthier sections of society and the ruling strata of the United States and its G-7 allies, in a framework that now incorporates their counterparts in the G-20, including the BRICS. However, even if one were to concede that have been some shifts in power at the global level it would be highly unlikely to result in changes in the basic political economy frameworks that underpin the governance of global health. For example, a close look at the BRICS enables dismissal of the idea that they are significantly influencing global health as a unified political bloc, despite evidence of some cooperation on finance and in health, with one or two BRICS countries supporting specific health initiatives. Nor are they threatening the frameworks of market-based neoliberal governance based upon public-private partnerships in delivering medical and health systems. The truth of this latter reality is partly reflected in Kickbusch’s points concerning the emerging trend towards “smart investment” and “commercial diplomacy” on the part of donors so that their pharmaceutical firms can benefit from a “bonanza” in supplying to new markets such as China, the biggest economy of the BRICS.^1^ Furthermore, if one looks closely at the new international trade and investment agreements – the ones that go beyond the Nairobi agreement (mentioned by Kickbusch) – what is striking is how far the BRICS and other poorer countries either go along with or actively support much greater marketization, liberalization, and privatization of goods and services, stronger guarantees for private property rights (including intellectual property rights agreements governing private ownership of patents for drugs and pharmaceutical products) thereby endorsing wider freedoms for large corporations. This allows corporations to press for greater commodification of healthcare and other social provisions, in ways consistent with the market civilization model outlined above.

## New Financial Systems

Our second criticism, therefore, relates to her optimism based on new financial institutions with shifts in donor strategies and on rapid economic growth in Africa and other poor countries. We consider such optimism to be misplaced for several reasons: (1) The relevance of the economic growth of poor countries has been overplayed (given that Africa accounts for about 20% of the world’s population but only 5%-6% of global gross domestic product [GDP])^25^ and there is a looming famine in Nigeria, Africa’s biggest economy; (2) The mal-distribution of that growth results in little improvement for the majority; (3) Associated corruption brings (in Africa and world wide) as many or more problems than it solves^26,27^; (4) As noted above, and reflecting a wider global pattern, the net flow of resources for many years has been predominantly from the poor to the wealthy^28^; and (5) The dominant model of development is built on intensification of exploitation and has created both great wealth and rising inequalities, while neglecting equity and sustainability.^29,30^

By contrast, we have noted elsewhere that it is not impossible to imagine or to identify alternatives to these dominant models, since democratic, typically grass-roots organisations, premised upon sustainable forms of healthcare and production of food and methods of livelihood, are actually asserting themselves in action in various parts of the world:

“*New forces are discernible in Latin America, more incipiently across Europe and indeed, in many parts of the world. They reflect new concepts of leadership, understood as involving a plurality of diverse forces in a collective movement – with hundreds of thousands, perhaps millions of intellectuals providing it with new concepts, organisational potentials and a multiple forms of leadership.*”^16^

## Global Health and Global Health Governance are Politics Writ Large

Thirdly, while Kickbusch is correct that the challenges ahead are deeply political she fails to address fundamental ways in which power, power structures and dynamics and the nature of actually existing capitalism and its links to global health could and should be addressed in order to be able to think about the future in a basically new way.^18^ Indeed, she makes no mention of the fact that the problems we face today are also the legacy of centuries of imperialism and colonialism, and of ideological and political agendas that have long been recognised as flawed, and show little signs of abating.^31^ The more recent problems for health arising from neoliberalism are highlighted in the Lancet-University of Oslo Commission Report,^16^ but even there, basic underlying structures of power, wealth and global inequality, and their effects on agency and governance, are not fully accounted for.

This is why we are sceptical of her idea that “commercial diplomacy and new investment strategies” will reshape global health governance in a beneficial way or indeed, that current new strategic thinking will be adequate to rectify serious deficiencies. All of the above observations articulated several decades ago, and oft repeated in a variety of ways, seem to have little effect on the unfolding of major global crises. For example, the responses from the United Nations (UN) and wealthy countries to the recent Ebola crisis have amounted to little more than recommendations for augmented philanthropy to build more resilient public health systems that could contain future outbreaks.^32-34^ In our view, the recently successful UN meetings while necessary are insufficient and unlikely to lead to viable and successful progress within the contemporary paradigms of power as they are actually practiced globally.^35^

Kickbusch’s proposal that global health has the goal of “equitable access to health in all regions of the globe” seems like wishful thinking given that 70% of the world population lives on less than $10 per day.^36^ This goal would only be remotely possible with an entirely differently structured global political economy involving understanding of and measures to address the interface between the ecological crisis, the financial crisis and the crisis of social dislocation, or what we have called elsewhere the global organic crisis.^13^ The health of people can no longer be seen as separate from the health of the planet^37,38^

and wealth measured by growth of GDP is not the only way to improve global health.^39^

## Reviews of Global Health Governance

Our final cautionary note is a reminder that an extensive review of the principal concerns of global leadership and global governance, following the 2008 meltdown of global capitalism, concluded that its priorities were to: (1) Sustain global capitalism; (2) Enlarge market civilization and accelerate its potentials for accumulation of capital; and (3) Maintain a condition of de-politicization, quiescence and political apathy amongst the population, or else to channel and incorporate forms of resistance to prevent political contestation on the nature and purposes of the political economy and practices of rule. By contrast little has been done to address the fundamental and highly negative impacts of the financial crisis on the basic livelihoods, life chances and health of the vast majority of the world’s population.^13^

A further key political point concerns the idea of the “rise of the rest,” a slogan coined by a member of the US foreign policy establishment. However, it reflects what philosophers call the *fallacy of misplaced concreteness*, that is the reification of the structures and processes of power that actually exist in the world. These continue to rest on concentrated control of capital in state formations that are either largely authoritarian or undergoing a crisis of representative democracy.^40^ These structures and developments are connected to struggles over the intensified exploitation of human beings, non-renewable resources, and biosphere.^41^

We have suggested elsewhere that an alternative paradigm of progress should not be based upon “sustainable development” as we consider this to be an oxymoron that allows for prevailing patterns of capitalist development to continue. By contrast we think an alternative concept of progress grounded in an understanding of historical structures, political economy and ecologically responsible health ethics is sorely needed to address global health governance challenges, one that we would argue should be premised upon global solidarity and the “development of sustainability.”^35^

## Ethical issues

Not applicable.

## Competing interests

Authors declare that they have no competing interests.

## Authors’ contributions

SG and SB wrote the first draft and worked closely together on subsequent versions and the corrections in response to reviewers.

## Authors’ affiliations

^1^Department of Political Science, York University, Toronto, ON, Canada. ^2^University of Cape Town, Cape Town, South Africa. ^3^Dalla Lana School of Public Health, University of Toronto, Toronto, ON, Canada.
